# Ocular Complications in Patients With Transfusion‐Dependent Beta‐Thalassemia Receiving Deferasirox: A Cross‐Sectional Study

**DOI:** 10.1002/hsr2.73053

**Published:** 2026-08-17

**Authors:** Abdolreza Medghalchi, Azadeh Rajabzadeh Kanafi, Yousef Alizadeh, Adel Baghersalimi, Maryam Doorandish, Afagh Hassanzadeh Rad, Shila Kianmehr, Bahram Darbandi

**Affiliations:** ^1^ Department of Eye, Eye Research Center, Amiralmomenin Hospital Guilan University of Medical Sciences Rasht Iran; ^2^ Pediatric Diseases Research Center Guilan University of Medical Sciences Rasht Iran

**Keywords:** beta‐thalassemia, deferasirox, ocular complication, visual acuity

## Abstract

**Background and Aims:**

Transfusion‐dependent beta‐thalassemia is a chronic hematologic disorder necessitating lifelong blood transfusions and iron chelation therapy, which can lead to systemic and ocular complications. This study aimed to assess the frequency and characteristics of ocular complications in beta‐thalassemia patients treated with deferasirox.

**Methods:**

In this cross‐sectional study, 75 patients with transfusion‐dependent beta‐thalassemia receiving deferasirox for at least 1 year were evaluated. Demographic and clinical characteristics, including hemoglobin (Hb) and ferritin levels over 6 months, were collected. Comprehensive ophthalmological assessments included best corrected visual acuity (BCVA), refraction, slit‐lamp, fundus examination, optical coherence tomography (OCT), and fundus autofluorescence (FAF). Data were analyzed using SPSS version 26, and the significance level was set at 0.05.

**Results:**

The mean age of the 75 patients was 27.8 ± 8.3 years, and 69.3% were female. The mean duration of deferasirox treatment was 53.6 ± 33.3 months at a daily dose of 24.1 ± 7.99 mg/kg. Mean Hb and ferritin levels were 8.99 ± 2.12 g/dL and 1419.8 ± 1218.6 ng/mL, respectively. Cataracts were identified in 7 of 75 patients (9.3%), with bilateral involvement in all affected patients. Ophthalmologic measurements were analyzed separately for each eye. BCVA showed a positive correlation with Hb in the right eye (*ρ* = 0.460, *p* < 0.001). Mean myopic refractive errors were −0.95 D in the right eye and −0.83 D in the left eye. With‐the‐rule astigmatism was the most frequent subtype, accounting for 68% of evaluated eyes. FAF, performed in 43 patients, identified angioid streaks and hyperautofluorescent changes. A significant association was observed between cylindrical refractive error and treatment duration in the left eye (*p* = 0.010), and between cylindrical refractive error and daily deferasirox dose in the right eye (*p* = 0.020).

**Conclusion:**

Ocular abnormalities were observed in patients with transfusion‐dependent beta‐thalassemia receiving deferasirox therapy. Longitudinal controlled studies are required to clarify the contribution of deferasirox to these findings.

## 1 Introduction

Beta‐thalassemia is one of the most common inherited hemoglobinopathies worldwide, characterized by reduced or absent synthesis of the β‐globin chains of hemoglobin. The transfusion‐dependent form of the disease requires lifelong regular blood transfusions and iron chelation therapy to manage chronic anemia and prevent iron overload [1, 2]. Deferasirox is a widely used oral iron chelator for patients with transfusion‐dependent beta‐thalassemia, offering comparable efficacy to desferrioxamine, though concerns remain regarding its potential ocular toxicity and long‐term safety [3, 4]. While advances in treatment have significantly improved life expectancy and quality of life for patients with transfusion‐dependent beta‐thalassemia, these interventions are not without complications. Among the less frequently highlighted but clinically significant complications are ocular complications, which can impair visual function and impact daily activities [5, 6, 7, 8].

The presence of ocular abnormalities in patients with transfusion‐dependent beta‐thalassemia has been reported to range from 10% to over 80%, depending on the population studied, diagnostic methods, and treatment regimens [7, 8]. These abnormalities may include lenticular opacities, retinal pigment epithelium (RPE) degeneration, optic neuropathy, retinal vascular tortuosity, and visual acuity disturbances [9, 10]. Factors contributing to ocular involvement in transfusion‐dependent patients with beta‐thalassemia are multifactorial, including chronic anemia, iron overload, and the toxic effects of iron chelators such as desferrioxamine and deferasirox [9, 11]. Risk factors for ocular complications in this population include duration and intensity of iron chelation therapy, serum ferritin levels, hemoglobin levels, and overall disease duration. Additionally, the cumulative burden of transfusion‐related iron overload may lead to deposition of iron in ocular tissues, exacerbating the risk of structural and functional eye damage [8, 12, 13, 14]. Despite these risks, ocular assessments are often underutilized in routine care, and early changes may go undetected until visual symptoms manifest.

Notably, while desferrioxamine has been historically associated with retinal and optic nerve toxicity, emerging reports have also raised concerns about the ocular safety profile of newer oral chelators [15, 16]. Given the potential for irreversible visual impairment, understanding the burden and characteristics of ocular abnormalities in these patients is crucial. It is noteworthy that early detection and management of visual disorders in these patients are essential, particularly as some ocular changes may be subtle or asymptomatic in early stages. This study investigated the frequency and spectrum of ocular findings in patients with transfusion‐dependent beta‐thalassemia, considering their correlation with hematologic and chelation parameters.

## 2 Methods

### 2.1 Study Design and Patients' Characteristics

This cross‐sectional study included 75 patients with transfusion‐dependent beta‐thalassemia who were receiving deferasirox and referred to the 17‐Shahrivar Pediatric Hospital and the Besat Clinic in Rasht, Iran, in 2021. We obtained informed consent from patients' guardians. The sample size was calculated based on the study by Golpayegani et al. and by considering a 10% dropout rate [17] using the following formula:

$$n=\frac{Z^{2}\mathrm{pq}}{d^{2}}$$

$$1-\alpha =\%95\;{Z\;}_{1-\frac{\alpha }{2}}=1.96P\;=\;16\%$$

$$n0=\frac{\left(({{Z\;}_{1-\frac{\alpha }{2}})}^{2}\right)\times P\;\left(1-p\right)}{d^{2}}=\frac{1.96^{2}\times 0.16\times (1-0.16)}{0.05^{2}}$$

*n* = *n*0/1 + (*n*0 − 1/*N*)

*n* = 67.54

Participants were recruited consecutively using census sampling to reduce selection bias. Patients with incomplete ophthalmologic evaluations were excluded from the final analysis. Inclusion criteria were: a confirmed diagnosis of transfusion‐dependent β‐thalassemia and continuous treatment with deferasirox for at least 1 year. According to the age of patients, they were categorized into two groups (< 28 years and ≥ 28 years). Exclusion criteria included pre‐existing ocular conditions such as aphakia, strabismus, a history of ocular surgery or trauma, or any retinal disease. Furthermore, patients who did not attend the scheduled ophthalmological examinations were excluded from the final analysis. Informed consent was obtained from all participants before enrollment in the study. The current study was confirmed by the ethical committee of the Guilan University of Medical Sciences (IR.GUMS.REC.1400.560).

### 2.2 Data Collection

After obtaining medical histories, demographic, clinical, and laboratory data of patients, including age, gender, duration of deferasirox therapy, dosage, and the mean serum ferritin and hemoglobin (Hb) levels over 6 months were recorded.

### 2.3 Retinal Evaluation

All ophthalmologic examinations were performed by a single experienced ophthalmologist to minimize interobserver variability. The assessments included measurement of best corrected visual acuity (BCVA) using a Snellen chart [18, 19] and reported in logarithm of the minimum angle of resolution (logMAR) units, refraction testing using an autorefractometer (expressed in diopters [D]), and anterior segment evaluation via slit‐lamp biomicroscopy. Cataracts, if present, were classified into anterior subcapsular, posterior subcapsular, cortical, or nuclear types.

Astigmatism was categorized based on the axis of the negative cylinder as follows: with‐the‐rule (axis 180° ± 20°), against‐the‐rule (axis 90° ± 20°), and oblique (axis between 21°–69° or 111°–159°). Posterior segment examination was performed using indirect ophthalmoscopy following pupil dilation (minimum diameter of 5 mm).

Retinal and choroidal assessments were conducted using optical coherence tomography (OCT) and fundus autofluorescence (FAF). OCT imaging was performed using the Spectralis device (Heidelberg Engineering GmbH, Heidelberg, Germany), which employs a low‐coherence superluminescent diode source based on Michelson interferometry principles [20, 21]. FAF imaging was performed in 43 patients due to the limited device availability and patient cooperation during imaging sessions.

Macular spectral‐domain OCT (SD‐OCT) was acquired using both a high‐resolution 9 mm horizontal and vertical line scan passing through the center of the fovea (line scan protocol) and a 6 × 6 mm volume scan protocol consisting of 25 lines centered on the fovea. If the 6 × 6 mm volume scan revealed any abnormalities in the parafoveal region, the area was further evaluated with additional high‐resolution 9 mm horizontal and vertical scans.

Choroidal assessment by OCT was classified as either normal or abnormal. Retinal structural integrity was evaluated in both the inner and outer retinal layers. Inner retinal abnormalities were defined as disruptions in the internal limiting membrane, nerve fiber layer, ganglion cell layer, inner plexiform layer, or inner nuclear layer. Outer retinal damage included alterations in the external limiting membrane, photoreceptor inner and outer segments (rods and cones), RPE, and Bruch's membrane.

FAF imaging (55° × 55°, centered on the macula) was performed using the Spectralis device (Heidelberg Engineering GmbH, Heidelberg, Germany). FAF images were evaluated to assess retinal autofluorescence patterns and categorized as either normal or abnormal according to predefined criteria [22, 23].

### 2.4 Statistical Analysis

Continuous variables were summarized as mean ± standard deviation (SD) for normally distributed data and median (interquartile range [IQR]) for non‐normally distributed variables. Categorical variables were presented as frequencies and percentages. Normality of continuous variables was assessed using the Kolmogorov–Smirnov test. Comparisons between groups were performed using independent samples *t*‐test or Mann–Whitney U test as appropriate. Demographic and hematologic variables were analyzed at the patient level (*n* = 75), whereas ophthalmologic variables were analyzed separately for the right and left eyes. Because measurements from fellow eyes are not statistically independent, right‐ and left‐eye data are presented separately, and no pooled eye‐level analyses were performed. Categorical variables were compared using *χ* ^2^ or Fisher's exact test. Spearman's rank correlation coefficient (*ρ*) was used to evaluate correlations between clinical and ophthalmologic variables. All statistical tests were two‐sided. Statistical analyses were performed using SPSS software version 26 (IBM Corp., Armonk, NY, USA). All tests were two‐sided, and a *p* value < 0.05 was considered statistically significant.

## 3 Results

Out of 75 patients, 52 were females (69.3%), and the mean age of the patients was 27.8 ± 8.3 years (13–45 years). The mean body weight was 56.01 ± 8.13 kg.

### 3.1 Hematologic Findings

The mean duration of deferasirox use was approximately 53.6 ± 33.3 months, and the mean daily dosage was 24.1 ± 7.99 mg/kg. Over the past 6 months, the mean Hb and ferritin levels were 8.99 ± 2.12 g/dL and 1419.8 ± 1218.6 ng/mL, respectively.

### 3.2 Ophthalmologic Findings

The mean BCVA was 0.008 ± 0.030 in both the right and left eyes (range: 0.000–0.150). Cataracts were identified in 7 of 75 patients (9.3%), with bilateral involvement in all affected patients. Cataract prevalence was slightly higher among patients aged < 28 years compared with those aged ≥ 28 years (10.8% vs. 7.9%, respectively) and among females compared with males (11.5% vs. 4.3%, respectively); however, these differences were not statistically significant (age comparison: *p*= 0.711; gender comparison: *p*= 0.427) (Table 1).

**Table 1:** Frequency of cataract and mean BCVA based on age and gender in beta‐thalassemia patients receiving deferasirox.

Variable		Cataract	Right eye	Left eye	Cataract	Right eye
			*n* (%)	*p* value	*n* (%)	*p* value
Age	< 28 years	Absent	33 (89.2%)	0.711	33 (89.2%)	0.711
		Present	4 (10.8%)		4 (10.8%)	
	≥ 28 years	Absent	35 (92.1%)		35 (92.1%)	
		Present	3 (7.9%)		3 (7.9%)	
Gender	Female	Absent	46 (88.5%)	0.427	46 (88.5%)	0.427
		Present	6 (11.5%)		6 (11.5%)	
	Male	Absent	22 (95.7%)		22 (95.7%)	
		Present	1 (4.3%)		1 (4.3%)	

Abbreviations: BCVA, best corrected visual acuity; SD, standard deviation.

Mean BCVA values were comparable between age groups and genders. In the right eye, the mean BCVA was 0.009 ± 0.035 in participants aged < 28 years and 0.006 ± 0.027 in those aged ≥ 28 years (*p *= 0.650). In the left eye, the corresponding values were 0.035 ± 0.017 and 0.003 ± 0.003, respectively (*p *= 0.080). Female participants showed slightly higher BCVA values than males in both eyes, although the differences were not statistically significant (Table 2).

**Table 2:** Frequency of cataract and mean BCVA based on age and gender in beta‐thalassemia patients receiving deferasirox.

Variable		BCVA			
		Right eye		Left eye	
		(Mean ± SD)	*p* value	(Mean ± SD)	*p* value
Age	< 28 years	0.009 ± 0.035	0.650	0.035 ± 0.017	0.080
	≥ 28 years	0.006 ± 0.027		0.003 ± 0.003	
Gender	Female	0.010 ± 0.005	0.390	0.024 ± 0.013	0.440
	Male	0.003 ± 0.003		0.009 ± 0.006	

Abbreviations: BCVA, best corrected visual acuity; SD, standard deviation.

Correlation analysis demonstrated a moderate positive association between Hb levels and BCVA in the right eye (Spearman's *ρ *= 0.460, *p*< 0.001). In contrast, treatment duration, deferasirox daily dose, and ferritin levels showed weak correlations with BCVA in either eye, none of which reached statistical significance. Patients without cataracts had a longer mean duration of treatment (54.8 ± 34.3 months vs. 41.1 ± 19.4 months) and slightly higher Hb levels (9.02 ± 2.22 g/dL vs. 8.81 ± 0.52 g/dL) compared with those with cataracts; however, these differences were not statistically significant (treatment duration: *p*= 0.372; Hb: *p*= 0.942). Similarly, no significant differences were observed regarding daily deferasirox dose or ferritin levels between the two groups (Table 3).

**Table 3:** Correlation of BCVA with quantitative factors in beta‐thalassemia patients receiving deferasirox.

Variable (mean ± SD)	OD and OS cataract			BCVA correlation*			
	Absent	Present	*p* value	Right eye	*p* value	Left eye	*p* value
Treatment duration (months)	54.8 ± 34.26	41.14 ± 19.42	0.372	−0.048	0.686	−0.089	0.449
Daily dose (mg/kg)	24.05 ± 8.21	24.57 ± 5.94	0.848	−0.046	0.069	−0.207	0.075
Hemoglobin (past 6 months)	9.02 ± 2.22	8.81 ± 0.52	0.942	0.460	< 0.001	−0.150	0.890
Ferritin (past 6 months)	1400 ± 1140.12	1554.28 ± 1983.27	0.402	−0.038	0.840	−0.093	0.420

*Note:* Correlation (Spearman's *ρ*). *Correlation (Spearman's ρ).

Abbreviations: BCVA, best corrected visual acuity; SD, standard deviation.

Refractive errors, including myopia, hyperopia, and astigmatism, were analyzed separately for each eye. Mean myopic refractive error was −0.95 ± 0.11 D in the right eye and −0.83 ± 0.10 D in the left eye. Mean hyperopic refractive error was 0.62 ± 0.25 D and 0.56 ± 0.18 D in the right and left eyes, respectively. Mean cylindrical refractive error was −0.72 ± 0.50 D in the right eye and −0.71 ± 0.068 D in the left eye. No statistically significant differences in refractive error patterns were observed according to age or gender. Older adults (≥ 28 years) demonstrated slightly greater mean myopic and cylindrical refractive errors, whereas younger participants (< 28 years) exhibited somewhat higher hyperopic refractive values. Female participants also showed marginally higher mean myopic refractive errors than males; however, all comparisons remained statistically non‐significant (Table 4).

**Table 4:** Mean spherical (myopia and hyperopia) and cylindrical refractive errors among age groups and genders in beta‐thalassemia patients receiving deferasirox.

Variable		Spherical and cylindrical refractive errors Mean ± SD					
		Myopia		Hyperopia		Cylinder	
		Right eye	Left eye	Right eye	Left eye	Right eye	Left eye
Age	< 28 years	−0.87 ± 0.13 D	−0.79 ± 0.13 D	0.90 ± 0.46 D	0.78 ± 0.30 D	−0.65 ± 0.08 D	−0.65 ± 0.01 D
	≥ 28 years	−1.01 ± 0.16 D	−0.86 ± 0.16 D	0.30 ± 0.10 D	0.32 ± 0.13 D	−0.78 ± 0.09 D	−0.76 ± 0.08 D
	*p* value	0.540	0.740	0.220	0.200	0.300	0.440
Gender	Female	−0.96 ± 0.13 D	−0.87 ± 0.13 D	0.73 ± 0.42 D	0.29 ± 0.06 D	−0.81 ± 0.089 D	−0.66 ± 0.07 D
	Male	−0.91 ± 0.19 D	−0.72 ± 0.15 D	0.47 ± 0.15 D	0.95 ± 0.41 D	−0.53 ± 0.08 D	−0.79 ± 0.14 D
	*p* value	0.860	0.530	0.610	0.070	0.450	0.370

Abbreviations: D, diopter; SD, standard deviation.

Correlation analyses revealed a weak positive association between duration of deferasirox use and cylindrical refractive error in the left eye (*ρ *= 0.294, *p*= 0.010). A borderline association was also observed between treatment duration and myopic refractive error in the left eye (*ρ *= 0.110, *p*= 0.050). In addition, higher daily deferasirox doses were weakly correlated with greater cylindrical refractive error in the right eye (*ρ *= 0.260, *p*= 0.020), whereas an inverse borderline association was observed between daily dose and hyperopic refractive error in the right eye (*ρ *= −0.300, *p*= 0.050) (Table 5).

**Table 5:** Correlation of spherical (myopia and hyperopia) and cylindrical refractive errors with clinical variables in beta‐thalassemia patients receiving deferasirox.

Variable		Myopia		Hyperopia		Cylinder	
		Right eye	Left eye	Right eye	Left eye	Right eye	Left eye
Duration of use (months)	Correlation	0.100	0.110	−0.100	−0.100	0.100	0.294
	*p* value	0.540	0.050	0.520	0.540	0.390	0.010
Daily dose	Correlation	−0.070	−0.100	−0.300	−0.090	0.260	0.198
	*p* value	0.680	0.530	0.050	0.580	0.020	0.089
Hemoglobin (past 6 months)	Correlation	0.130	−0.160	−0.054	−0.0970	−0.039	0.010
	*p* value	0.420	0.310	0.740	0.570	0.730	0.900
Ferritin (past 6 months)	Correlation	0.240	0.290	−0.080	−0.055	0.020	0.132
	*p* value	0.150	0.070	0.610	0.750	0.086	0.260

*Note:* Correlation (Spearman's *ρ*).

Abbreviations: OD, right eye; OS, left eye.

OCT findings were assessed at the eye level. OCT identified one case of bilateral vitreous degeneration, one case of bilateral VMA, one case with a subtle ellipsoid zone defect in the central macula, and one case of pigment epithelial detachment (PED) in the right eye. FAF imaging was performed in 43 patients (86 eyes when bilateral imaging was available) and identified two cases of angioid streaks, two cases of localized hyperautofluorescent lesions, and one additional case with unspecified hyperautofluorescent changes, suggesting retinal pigment epithelium and outer retinal abnormalities.

**Figure 1:**
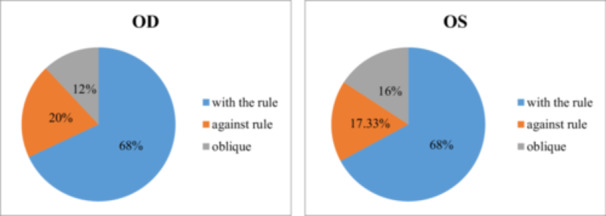
Distribution of astigmatism subtypes among right (OD) and left (OS) eyes in patients with transfusion‐dependent beta‐thalassemia receiving deferasirox. With‐the‐rule astigmatism was the predominant subtype in both eyes.

Analysis of astigmatism patterns showed that with‐the‐rule astigmatism was the predominant subtype, accounting for 68% of evaluated eyes bilaterally. Against‐the‐rule astigmatism was observed in 20.0% of right eyes and 17.3% of left eyes, while oblique astigmatism accounted for 12.0% and 16.0% of right and left eyes, respectively. Mean cylindrical power was −0.62 D and −0.64 D for with‐the‐rule astigmatism, −1.06 D and −1.01 D for against‐the‐rule astigmatism, and −0.50 D and −0.67 D for oblique astigmatism in the right and left eyes, respectively (Figure 1).

## 4 Discussion

The shortened life expectancy in thalassemia patients is primarily due to complications such as liver failure and cardiomyopathy caused by chronic iron overload from frequent blood transfusions, making iron chelation therapy, such as deferasirox, essential despite its potential side effects [3, 24, 25]. Evidence showed that deferasirox is an effective and generally well‐tolerated oral iron chelator for managing transfusion iron overload in patients with conditions such as beta‐thalassemia, but requires regular monitoring to manage potential long‐term adverse effects [26, 27].

The mean age of patients in our study was about 28 years, with the majority of females, which aligns closely with recent research on transfusion‐dependent beta‐thalassemia patients receiving deferasirox. Haghpanah et al. reported a similar mean age of approximately 28.5 years in their cohort, reflecting that advances in chelation therapy have improved patient survival into adulthood, and most of their patients were females [28]. In contrast, Aidenloo et al. found a lower mean age of about 18 years and a higher prevalence of male gender among thalasemia major patients with ocular complications [29]. This difference may be attributable to regional demographic variations or sample selection biases.

We also observed a frequency of 9.3% for cataract among patients receiving deferasirox. A study by Nuzzi et al. found that iron chelation therapy in patients with thalassemia major is not significantly associated with serious ocular complications; however, prolonged use of deferoxamine was linked to increased risk of presbyopia and reduced intraocular pressure, while deferiprone showed a possible protective effect against presbyopia [11]. Another study by Heydarian et al. showed that the incidence of cataract in thalassemia can range from 6.3% to 45%, indicating a high burden likely influenced by iron overload and iron chelation therapy [14]. While some studies suggest chelators may have a protective role by reducing iron‐induced oxidative stress, a key mechanism in cataractogenesis, others have raised concerns about the potential cataractogenic effects of certain agents, such as deferiprone [30, 31, 32].

Therefore, the current evidence regarding deferasirox‐associated ocular toxicity remains inconclusive, and interpretation of ocular findings should consider the multifactorial pathophysiology of beta‐thalassemia rather than attributing abnormalities solely to chelation therapy. Nonetheless, cataracts have also been observed in patients not receiving chelation, suggesting that chelation alone may not be the primary cause. The reported BCVA for left and right eyes showed no significant differences in this study. Evidence demonstrated that patients with beta‐thalassemia have reduced visual acuity, which results in more ocular complications [33, 34]. A study by Biag et al. observed that the prevalence of ocular complications in children aged 11–17 years old with beta thalassemia major was 22.7%, with the majority being male gender [10].

Furthermore, we found a positive correlation between Hb levels and BCVA in the right eye, indicating better visual acuity in patients with higher Hb concentrations. However, no statistically significant associations were found between BCVA and ferritin, treatment duration, or deferasirox dose in different genders and ages. Ocular abnormalities in severe anemia and thalassemia arise primarily from impaired oxygen supply to the eye tissues, leading to vascular damage and retinal ischemia. This hypoxic stress can cause hemorrhages, cotton wool spots, optic disc swelling, and edema, as well as disrupt normal retinal function. The resulting microvascular changes and inflammation increase the risk of vision impairment and other ocular complications associated with low levels of Hb [8, 35, 36].

The most prevalent refractive error in our study was astigmatism, particularly with‐the‐rule astigmatism, consistent with the general population trends. However, we found statistically significant correlations between cylindrical refractive errors and both the duration of deferasirox use in the left eye and the daily dose for the right eye. Evidence illustrated that a considerable population of thalassemia patients experienced astigmatism‐related problems [37]. A study by Koochakzadeh et al. identified astigmatism as a significant ocular complication in patients with thalassemia, with the most common type being against‐the‐rule astigmatism [38]. In another study by Naseem et al., it has been reported that of the thalassemia patients exhibited high prevalence of myopic astigmatism in the right and left eyes, while in the left eye, hyperopic astigmatism had a lower frequency [39].

In the current study, the marginally significant negative correlation between deferasirox dose and hyperopia in the right eye is intriguing and might reflect early structural lens or axial length alterations. Whether these changes are drug‐induced or related to thalassemia‐related growth and development remains speculative. Nuzzi et al. reported that iron chelation therapy in thalassemia major patients is generally not linked to severe visual function impairments, though limiting deferoxamine use may reduce ocular complications, with deferiprone and deferasirox potentially preferred, especially in patients over 40 years old [11].

Additionally, advanced retinal imaging using OCT and FAF in our study revealed several subtle yet clinically significant abnormalities, including VMA, ellipsoid zone disruption, and PED. FAF further highlighted angioid streaks and hyperautofluorescent areas, indicative of chronic RPE stress potentially driven by iron overload or oxidative damage related to chelation therapy. These findings align with previous studies describing tigroid patches, foveolar darkening, and RPE granularity in transfusion‐dependent beta‐thalassemia patients [13, 40]. The observed angioid streaks may reflect systemic connective tissue alterations or vascular fragility linked to iron dysregulation. Moreover, lenticular opacities and RPE degeneration have been correlated with desferrioxamine and deferiprone use, respectively [9]. While deferoxamine is commonly implicated in retinal toxicity, emerging evidence, including our observations, suggests that deferasirox may also carry subtle retinal risks [9, 41, 42, 43].

Ocular findings detected by OCT and FAF in thalassemia patients are variable and depend on individual patient conditions; however, these two imaging modalities are effective tools for identifying and diagnosing ocular abnormalities in this population. Moreover, Ocular manifestations in beta‐thalassemia are likely multifactorial and may reflect the combined effects of chronic anemia, iron overload, cumulative transfusion burden, and iron chelation exposure [13, 44]. The observed refractive abnormalities may represent incidental findings and should not necessarily be interpreted as clinically significant deferasirox‐related complications.

Previous investigations have reported minimal or no clinically significant retinal or lenticular toxicity associated with deferasirox therapy, particularly when standard therapeutic doses and regular ophthalmologic monitoring were applied [11, 45]. These studies suggested that many ocular abnormalities observed in transfusion‐dependent thalassemia patients may be more strongly related to chronic anemia, iron overload, oxidative stress, or cumulative transfusion burden rather than deferasirox exposure alone [13, 44]. Furthermore, some studies have concluded that deferasirox possesses a relatively favorable ocular safety profile compared with older chelators such as deferoxamine, for which retinal toxicity is more consistently documented [11, 42].

This study has several limitations. First, its cross‐sectional design precludes establishing temporal or causal relationships between deferasirox exposure and the observed ocular findings. Second, the relatively small sample size may have limited statistical power, particularly for subgroup and correlation analyses. In addition, the exploratory correlation analyses were not adjusted for multiple comparisons, increasing the risk of type I error. Third, the absence of a comparator group, such as patients receiving other iron chelators or no chelation therapy, limits attribution of the findings specifically to deferasirox. Furthermore, serum ferritin may not accurately reflect cumulative tissue iron burden because it is influenced by inflammation, infection, and liver dysfunction. Finally, because both eyes from the same patient were analyzed separately, intra‐subject correlation may have affected statistical independence. Therefore, the findings should be interpreted with caution. Prospective, controlled longitudinal studies with larger sample sizes are needed to clarify the independent contributions of deferasirox, iron overload, and β‐thalassemia to ocular abnormalities and to compare the ocular safety of different iron‐chelating therapies.

## 5 Conclusion

This cross‐sectional study identified several ocular abnormalities, including cataracts, refractive errors, and retinal imaging findings, among patients with transfusion‐dependent β‐thalassemia receiving deferasirox therapy. Although several treatment‐related characteristics were statistically associated with selected ophthalmic parameters, the observational design precludes any causal inference regarding the role of deferasirox.

## Author Contributions

**Abdolreza Medghalchi:** conceptualization, supervision, writing – original draft, writing – review and editing, methodology, investigation, project administration, resources. **Azadeh Rajabzadeh Kanafi:** data curation, investigation, formal analysis, writing – original draft, writing – review and editing. **Yousef Alizadeh:** investigation, writing – review and editing, methodology, writing – original draft, data curation. **Adel Baghersalimi:** conceptualization, methodology, data curation, validation, visualization, writing – original draft, writing – review and editing. **Maryam Doorandish:** investigation, writing – original draft, writing – review and editing, formal analysis, software. **Afagh Hassanzadeh Rad:** data curation, investigation, formal analysis, validation, writing – original draft. **Shila Kianmehr:** investigation, writing – original draft, writing – review and editing, visualization, formal analysis. **Bahram Darbandi:** conceptualization, methodology, data curation, investigation, validation, supervision, project administration, resources, writing – original draft, writing – review and editing.

## Funding

The authors have nothing to report.

## Ethics Statement

All methods of this study were carried out following relevant guidelines and regulations. Written informed consent was obtained from all participants. The study was approved by the ethical committee of the Guilan University of Medical Sciences, Rasht, Iran (IR.GUMS.REC.1400.560).

## Conflicts of Interest

The authors declare no conflicts of interest.

## Transparency Statement

The corresponding author, Bahram Darbandi, affirms that this manuscript is an honest, accurate, and transparent account of the study being reported; that no important aspects of the study have been omitted; and that any discrepancies from the study as planned (and, if relevant, registered) have been explained.

## Data Availability

The data that support the findings of this study are available from the corresponding author upon reasonable request.
